# 维奈克拉联合阿扎胞苷治疗难治复发性急性髓系白血病疗效及安全性分析

**DOI:** 10.3760/cma.j.issn.0253-2727.2021.10.012

**Published:** 2021-10

**Authors:** 李红 宗, 小霞 吴, 剑 张, 梦云 郦, 宝全 宋, 金玉 孔, 欣 孔, 晓慧 胡, 协炳 鲍, 惠英 仇, 德沛 吴

**Affiliations:** 苏州大学附属第一医院血液内科，江苏省血液研究所，国家血液系统疾病临床医学研究中心 215006 The First Affiliated Hospital of Soochow University, Jiangsu Institute of Hematology，National Clinical Research Center for Hematologic Diseases, Suzhou 215006

难治复发性急性髓系白血病（R/R AML）患者预后不佳，其长期存活率低于25％。传统的挽救性化疗缓解率较低，同时因患者原发耐药、药物累积的毒性作用、脏器功能衰退等原因限制了治疗方案的选择。近年来，靶向药物和去甲基化药物的应用，使部分R/R AML患者获得完全缓解（CR）状态，为后续桥接异基因造血干细胞移植提供可能。维奈克拉（VEN）是一种选择性小分子BCL-2抑制剂，已在临床前研究中显示出可诱导依赖BCL-2生存的恶性细胞凋亡。VEN联合去甲基化药物（HMA）或低剂量阿糖胞苷成为75岁及以上、不适合强化疗的初治AML患者的治疗新选择，能够使患者获得更长的总生存期和更高缓解率[1]–[2]。本研究对我中心36例应用VEN联合阿扎胞苷（AZA）方案治疗的R/R AML患者进行回顾性分析，探讨其临床疗效和安全性。

## 病例与方法

1. 一般资料：回顾性分析2018年10月26日至2021年3月8日苏州大学附属第一医院及苏州弘慈血液病医院收治的36例R/R AML患者的临床资料，诊断根据2016版WHO造血和淋巴组织肿瘤的分型诊断标准[3]。所有患者经骨髓细胞形态学、白血病免疫分型、细胞遗传学、分子生物学等检查确诊。36例R/R AML患者中，难治19例，化疗后复发11例，移植后复发6例。男18例，女18例，中位年龄41（18～74）岁。治疗前骨髓原始细胞比例的中位数为38.0％（4.5％～88.5％）。FLT3-ITD基因突变11例，DNMT3A基因突变8例，IDH1/2基因突变5例，PTPN11基因突变6例（表1）。按照2017年欧洲白血病网络（ELN）AML危险度分层体系，预后良好组4例，预后中等组13例，预后不良组19例。

**表1:** 36例难治复发性急性髓系白血病患者的临床特征

特征	数值
年龄［岁，*M*（范围）］	41（18～74）
用药前骨髓原始细胞［％，*M*（范围）］	38.0（4.5～88.5）
诊断［例（％）］	
难治	19（52.8）
复发	17（47.2）
性别［例（％）］	
男	18（50.0）
女	18（50.0）
预后分层［例（％）］	
低中危	17（47.2）
高危	19（52.8）
基因突变［例（％）］	
FLT3-ITD	11（30.6）
DNMT3A	8（22.2）
K/NRAS	7（19.4）
PTPN11	6（16.7）
NPM1	5（13.9）
IDH1/2	5（13.9）
TP53	5（13.9）
维奈克拉治疗周期［个，*M*（范围）］	1（1～3）
缓解状态［例（％）］	
CR	13（36.1）
CRi	2（5.6）
MLFS	1（2.8）
PR	2（5.6）
NR	18（50.0）
桥接移植［例（％）］	
CR后桥接移植	12
挽救性移植	8
脱离粒缺时间［d，*M*（范围）］	24（10～55）
达PLT>50×10^9^/L时间［d，*M*（范围）］	15（4～60）

注：CR：完全缓解；CRi：CR伴血液学不完全恢复；MLFS：形态学无白血病状态；PR：部分缓解；NR：未缓解

2. 治疗方法：VEN 100 mg第1天，200 mg第2天，400 mg第3～28天，口服,根据骨髓抑制情况和药物相互作用相应调整。AZA 75 mg/m^2^，第1～7天。其中10例因用药开始时即存在持续粒细胞缺乏（粒缺），VEN最大剂量降低为100～200 mg/d，这部分患者因粒缺同时采用伏立康唑预防真菌感染。后期用药过程中有22例患者因出现粒缺加用伏立康唑预防真菌感染，同时下调VEN剂量到100 mg/d。重度粒缺（<0.2×10^9^/L）合并重度感染时暂停口服VEN。患者最多可以接受3个周期的治疗。骨髓抑制期HGB<60 g/L或出现明显贫血症状时输注悬浮红细胞，PLT<20×10^9^/L或有明显出血倾向时输注血小板。口服用药第14天复查骨髓，形态学中原始细胞<5％，加用G-CSF，直至中性粒细胞绝对计数（ANC）>0.5×10^9^/L，且脱离血小板输注，VEN加量至200 mg/d或400 mg/d，停用伏立康唑。

3. 疗效和不良反应：完全缓解（CR）：骨髓原始细胞<5％，原始细胞内不含有Auer小体，无髓外白血病；外周血ANC>1.0×10^9^/L，且PLT>100×10^9^/L。CR伴血液学不完全恢复（CRi）：外周血ANC<1.0×10^9^/L或PLT<100×10^9^/L，其他满足CR的标准。形态学无白血病状态（MLFS）：白血病症状和体征消失，未见白血病细胞，骨髓原始粒细胞比例<5％，且未伴有髓外白血病。部分缓解（PR）：化疗后骨髓原始细胞比例减少至少50％，且下降到5％～25％，外周血细胞计数正常[4]。ORR定义为患者在治疗后达到CR、CRi、PR、MLFS的比例[5]。未缓解（NR）：诱导治疗后未获得CR或PR。总生存（OS）时间为开始应用VEN至患者死亡或末次随访的时间（失访时间）。无病生存（DFS）时间为从获CR起至患者复发或在CR期间发生死亡的时间。所有不良事件按照NCI常见不良事件术语标准4.0版进行报告和分级[6]。

4. 随访：以电话或短信方式随访，随访截止日期为2021年3月8日。

5. 统计学处理：采用SPSS 26.0软件进行统计学分析。计数资料以百分比（％）表示，采用卡方检验；生存分析采用Kaplan-Meier法，差异性检验采用Log-rank法。检验水准*α*＝0.05，*P*<0.05为差异有统计学意义。

## 结果

1. 疗效评价：中位随访9.0（0.6～24.2）个月，36例R/R AML患者接受VEN+AZA联合化疗的中位周期数为1（1～3）个，其中18例（50％）患者观察到客观反应，包括13例CR，2例CRi，1例MLFS，2例PR。11例患者存在FLT3-ITD基因突变，CR/CRi率为45.5％（4例获CR，1例获CRi）。5例患者存在IDH1/2基因突变，其中4例患者达CR，CR率为80％。PTPN11基因突变6例，1例达CR，CR率为16.7％。此外我们发现预后良好组、预后中等组与预后不良组的CR率差异无统计学意义（分别为75.0％、30.8％、36.1％，*P*>0.05）（图1）。

**图1:**
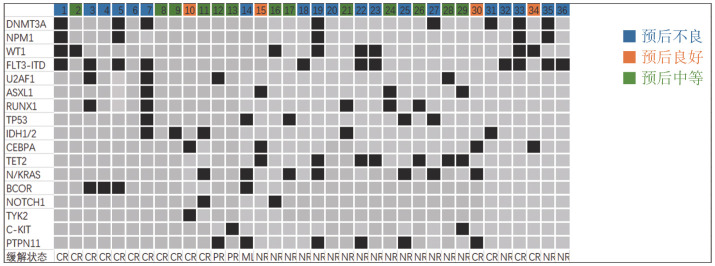
36例难治复发性急性髓系白血病患者基因突变情况及应用维奈克拉联合阿扎胞苷的疗效 CR：完全缓解；PR：部分缓解；NR：未缓解

36例患者6个月OS、DFS率分别为69.6％、48.9％，中位OS时间为11.5个月。20例患者接受异基因造血干细胞移植，均使用清髓预处理方案（改良BUCY）。8例非CR状态下行挽救性移植的患者在移植后第30天评估骨髓状态时有7例获得CR，但这7例患者中4例再次复发（4例复发患者缓解的时间分别为21、26、60、180 d，其余3例至随访截止日缓解的时间分别为6.4、9.9、15个月）。CR后桥接移植与非CR状态下行移植患者的6个月OS率分别为85.7％、62.5％（*P*＝0.160），其6个月的DFS率分别为83.3％、0（*P*＝0.001），CR后桥接移植组未达到中位OS时间，非CR状态桥接移植组中位OS时间为11.5个月。

2. 不良反应：截至2021年3月8日，36例患者中22例（61.1％）存活，12例（33.3％）死亡，2例（5.6％）失访。死亡原因包括疾病进展6例，复发6例。所有患者均有≥3级血液学不良反应，分别为血小板减少27例（75.0％）、中性粒细胞减少32例（88.9％）、贫血19例（52.8％），但没有出现致死性大出血。最常见的非血液学不良反应为肺部感染（27.8％）、恶心（19.4％）、呕吐（11.1％）。1例患者因重度粒缺合并感染暂停口服VEN。所有患者均未见肿瘤溶解综合征（TLS）。预后良好、预后中等组与预后不良组≥3级血液学不良反应及感染发生率差异均无统计学意义（血液学不良反应：100％对84.6％对84.2％，*P*>0.05；感染：25.0％对46.2％对15.8％，*P*>0.05）。

## 讨论

VEN是小分子BCL-2抑制剂，是一种具有广泛抗肿瘤活性的药物。BCL-2家族在细胞死亡信号通路中参与调控线粒体依赖的关键步骤，其是由促凋亡分子、抗凋亡分子、效应蛋白三个亚群间的蛋白与蛋白相互作用，主要负责调控内源性（线粒体）细胞凋亡信号通路[7]。在细胞应激的情况下，促凋亡蛋白BAX和BAK进入线粒体并且诱导细胞色素C释放，随后激活caspase级联反应，促进细胞凋亡[8]。VEN单药治疗R/R AML患者的CR+CRi率仅为19％[9]。VEN联合HMA方案具有较高的CR率，Lou等[10]的研究纳入48例R/R AML患者，联合用药的ORR可达到47.9％，中位OS时间为9.6个月。Aldoss等[11]的研究表明R/R AML患者接受联合用药的CR/CRi率为46％。二者之间可能存在协同作用：AZA既可以降低MCL-1水平，延缓VEN耐药性的出现，又能激活AML细胞中的BAX和线粒体凋亡，二者在体外协同杀伤白血病细胞，并在体内表现出联合抗肿瘤活性[12]。对于R/R AML患者，选择合适的再诱导化疗方案使患者获得CR以桥接异基因造血干细胞移植是一个挑战，本研究的患者接受VEN联合AZA方案的CR/CRi率与国外相似[10]–[11]。FLT3基因突变发生在25％～35％的AML患者中，通常与高复发风险和低OS率有关，有文献报道FLT3突变的R/R AML患者行VEN+HMA治疗的CR/CRi率为42％[13]。在我们的研究中，这类患者的CR/CRi率为45.5％。此外，具有IDH1/2突变的初治AML患者的CR/CRi率达78.5％[14]，而在我们的研究中，R/R AML患者也极大获益于VEN+HMA方案，其CR率可达80％。在安全性方面，有研究表明所有R/R髓系疾病患者接受VEN联合HMA/LDAC治疗时都出现了≥3级中性粒细胞减少，且≥3级感染发生率为72％[15]，而在我们的研究中，血液系统毒性及感染的发生率明显降低。

对于R/R AML患者，选择何种化疗方案从而提高AML患者生存时间一直是研究的重点。传统的治疗方案为以高剂量阿糖胞苷为主，联合蒽环类药物、氟达拉滨（FLAG）或克拉曲滨（CLAG）等，然而治疗过程中所有患者均会经历重度骨髓抑制，化疗相关并发症多，粒缺状态下感染发生率高。VEN+AZA方案具有良好的安全性和疗效，感染发生率低，本组病例的感染发生率为27.8％，明显低于传统二线强化疗方案[16]，而且具有较高的缓解率和较长的缓解持续时间。然而VEN在应用中存在耐药性，可能与下列因素有关：骨髓和外周血原始细胞比例低，单核细胞和中性粒细胞计数高，FAB分型中的M_4_、M_5_，KRAS、PTPN11、SF3B1基因突变[17]。

综上所述，本研究回顾性分析了36例R/R AML患者使用VEN+AZA方案的疗效以及安全性，结果显示联合用药缓解率高，OS时间长，且严重不良反应少，有望成为挽救性治疗的可行选择之一。但鉴于本研究纳入样本量少、随访时间短，所得结论还有待多中心、大规模临床研究确证。

## References

[1] DiNardo CD, Pratz KW, Letai A (2018). Safety and preliminary efficacy of venetoclax with decitabine or azacitidine in elderly patients with previously untreated acute myeloid leukaemia: a non-randomised, open-label, phase 1b study[J]. Lancet Oncol.

[2] DiNardo CD, Pratz K, Pullarkat V (2019). Venetoclax combined with decitabine or azacitidine in treatment-naive, elderly patients with acute myeloid leukemia[J]. Blood.

[3] Arber DA, Orazi A, Hasserjian R (2016). The 2016 revision to the World Health Organization classification of myeloid neoplasms and acute leukemia[J]. Blood.

[4] Cheson BD, Greenberg PL, Bennett JM (2006). Clinical application and proposal for modification of the International Working Group (IWG) response criteria in myelodysplasia[J]. Blood.

[5] Liu B, Guo Y, Deng L (2020). The efficacy and adverse events of venetoclax in combination with hypomethylating agents treatment for patients with acute myeloid leukemia and myelodysplastic syndrome: a systematic review and meta-analysis[J]. Hematology.

[6] Wilson C NCI Common Terminology Criteria for Adverse Events (CTCAE) v3.0 and v4.0.

[7] Shamas-Din A, Kale J, Leber B (2013). Mechanisms of action of Bcl-2 family proteins[J]. Cold Spring Harb Perspect Biol.

[8] Guerra VA, DiNardo C, Konopleva M (2019). Venetoclax-based therapies for acute myeloid leukemia[J]. Best Pract Res Clin Haematol.

[9] Konopleva M, Pollyea DA, Potluri J (2016). Efficacy and Biological Correlates of Response in a Phase II Study of Venetoclax Monotherapy in Patients with Acute Myelogenous Leukemia[J]. Cancer Discov.

[10] Lou Y, Shao L, Mao L (2020). Efficacy and predictive factors of venetoclax combined with azacitidine as salvage therapy in advanced acute myeloid leukemia patients: A multicenter retrospective study[J]. Leuk Res.

[11] Aldoss I, Yang D, Pillai R (2019). Association of leukemia genetics with response to venetoclax and hypomethylating agents in relapsed/refractory acute myeloid leukemia[J]. Am J Hematol.

[12] Jin S, Cojocari D, Purkal JJ (2020). 5-Azacitidine Induces NOXA to Prime AML Cells for Venetoclax-Mediated Apoptosis[J]. Clin Cancer Res.

[13] Aldoss I, Zhang J, Mei M (2020). Venetoclax and hypomethylating agents in FLT3-mutated acute myeloid leukemia[J]. Am J Hematol.

[14] Pollyea DA, Dinardo CD, Arellano ML (2020). Results of Venetoclax and Azacitidine Combination in Chemotherapy Ineligible Untreated Patients with Acute Myeloid Leukemia with IDH 1/2Mutations[J]. Blood.

[15] DiNardo CD, Rausch CR, Benton C (2018). Clinical experience with the BCL2-inhibitor venetoclax in combination therapy for relapsed and refractory acute myeloid leukemia and related myeloid malignancies[J]. Am J Hematol.

[16] Pastore D, Specchia G, Carluccio P (2003). FLAG-IDA in the treatment of refractory/relapsed acute myeloid leukemia: single-center experience[J]. Ann Hematol.

[17] Zhang H, Nakauchi Y, Köhnke T (2020). Integrated analysis of patient samples identifies biomarkers for venetoclax efficacy and combination strategies in acute myeloid leukemia[J]. Nat Cancer.

